# Effectiveness and Safety of Teduglutide Treatment in Adult Patients with Short Bowel Syndrome: A Case Series and Review of Current Evidence

**DOI:** 10.3390/jcm15052033

**Published:** 2026-03-06

**Authors:** Fotios Fousekis, Ioanna Nefeli Mastorogianni, Maria Tzouvala, Andreas Larentzakis, Eirini Zacharopoulou, Georgios D. Lianos, Konstantinos Mpakogiannis, Odysseas Tsakai, Alexandros Tzallas, Sotirios D. Georgopoulos, George Michalopoulos, Konstantinos H. Katsanos, Konstantinos Vlachos

**Affiliations:** 1 Department of Gastroenterology, School of Health Sciences, University of Ioannina, 45110 Ioannina, Greece; nefelimastorogianni@gmail.com (I.N.M.); kostismpakogiannis@gmail.com (K.M.); khkostas@hotmail.com (K.H.K.); 2 Gastroenterology Department, General Hospital Nikaia Piraeus ‘Agios Panteleimon’, 18454 Athens, Greece; tzouvalam@gmail.com (M.T.); eirinizachar@gmail.com (E.Z.); 3 Division of Surgical Anatomy, School of Medicine, European University Cyprus (EUC), Nicosia 2404, Cyprus; info@larentzakis.org; 4 Department of Surgery, School of Health Sciences, University of Ioannina, 45110 Ioannina, Greece; georgiolianos@yahoo.gr (G.D.L.);; 5 Department of Informatics & Telecommunications, School of Informatics & Telecommunications, University of Ioannina, Kostakioi, 47100 Arta, Greece; odysseas94@gmail.com (O.T.); atzallas@gmail.com (A.T.); 6 GI and Hepatology Department, Athens Medical, Paleo Faliron Hospital, 17564 Athens, Greece; georgpap@ath.forthnet.gr; 7 Department of Gastroenterology, Tzaneion General Hospital, 18536 Piraeus, Greece; gmicha78@hotmail.com

**Keywords:** short bowel syndrome, teduglutide, chronic intestinal failure, parenteral nutrition, GLP-2 analogue

## Abstract

**Background:** Short bowel syndrome (SBS) is the leading cause of chronic intestinal failure and is frequently associated with long-term dependence on parenteral nutrition (PN) and intravenous fluids. Teduglutide, a glucagon-like peptide-2 (GLP-2) analogue, promotes intestinal adaptation and has been demonstrated to reduce parenteral support requirements. However, real-world data from the Greek population are scarce. **Methods:** We conducted a non-interventional, multicenter, retrospective cohort study across 5 centers in Greece, including adult patients with SBS receiving teduglutide therapy. Demographic and clinical characteristics, parenteral nutrition and intravenous fluid requirements, body mass index (BMI), laboratory parameters, and adverse events were recorded at baseline and during follow-up at weeks 4, 12, 26, and 52. **Results:** Eight adult patients with SBS were included (75% female), with a median age of 53 years (range 19–71). Over 52 weeks of treatment, mean parenteral nutrition requirements decreased by approximately 45% compared with baseline (from 1430 to 788 kcal/day), while mean intravenous hydration requirements decreased by approximately 80% (from 5170 to 1000 mL/week). Complete independence from parenteral nutrition was achieved in 2 of 8 patients (25%). Nutritional status improved, with a 10.6% increase in mean BMI at Week 52. Teduglutide was generally well tolerated; mild adverse events occurred in 3 of 8 patients, were predominantly gastrointestinal, and did not lead to treatment discontinuation. **Conclusions:** This study provides data from the Greek population and supports the effectiveness and favorable safety profile of teduglutide in adult patients with SBS and chronic intestinal failure. Further prospective studies are warranted to better define predictors of response and optimize long-term management strategies.

## 1. Introduction

Short bowel syndrome (SBS) is a clinical condition resulting from the loss of a significant amount of the small intestine, most commonly due to extensive surgical resection. The syndrome is characterised by the malabsorption of macronutrients, micronutrients, water and electrolytes, which leads to symptoms such as diarrhoea, steatorrhea, dehydration, weight loss and malnutrition [1]. The severity of the condition and its clinical manifestations depend on the length and segments of intestine remaining, as well as on the presence or absence of the colon. The loss of specific regions, such as the terminal ileum, can result in vitamin B12 deficiency and bile acid malabsorption. SBS is the leading cause of chronic intestinal failure (IF), which is defined as the inability of the gut to absorb sufficient nutrients and fluids to maintain health without intravenous supplementation (parenteral nutrition) [2].

In adults, the leading causes of SBS include mesenteric ischaemia (infarction), Crohn’s disease, malignancy, trauma and complications from radiation enteritis [3]. In pediatric populations, the most common causes are congenital anomalies such as intestinal atresia and gastroschisis, as well as conditions such as necrotising enterocolitis and midgut volvulus [4]. Less commonly, SBS may result from non-resectional loss of intestinal function, such as severe motility disorders or mucosal diseases, but these are rare compared to anatomical loss [5]. 

The classification of SBS is primarily based on the anatomical configuration of the remaining intestine, as this has a direct impact on prognosis and management. SBS can be categorised in three types. Type 1 (end-jejunostomy) is characterised by the jejunum ending in a stoma with no continuity to the colon. This is the most severe form and is associated with substantial fluid and nutrient losses, as well as a high likelihood of chronic IF requiring long-term parenteral nutrition. Type 2 (jejunocolic anastomosis) involves an anastomosis between the jejunum and a portion of the colon, with an absence of the ileum and ileocecal valve. This configuration allows for some salvage of fluids and electrolytes from the colon and is associated with an intermediate prognosis. Type 3 (jejunoileocolic anastomosis) preserves the ileum, colon and ileocecal valve. This offers the most favourable prognosis and the greatest potential for intestinal adaptation, as well as the highest likelihood of weaning from parenteral nutrition [6].

The incidence of SBS in adults has remained low. Recent studies have reported annual incidence rates of between 0.6 and 5 cases per million adults, with prevalence estimates ranging from 0.12 to 2.74 per 100,000 adults worldwide [7]. However, SBS-associated hospitalisations have increased substantially, rising by 55% in the United States from 2005 to 2014 [8].

Nutritional support strategies are crucial in managing SBS. Initial stabilization often requires parenteral nutrition (PN) to address severe malabsorption, dehydration and electrolyte imbalances. The goal is to transition to enteral and oral nutrition as soon as possible, as this promotes intestinal adaptation and reduces PN-related complications [9]. Dietary interventions should be individualized, focusing on complex carbohydrates, moderate protein, and low fat, especially for patients with colon continuity [10]. For those unable to meet nutritional needs orally, enteral nutrition via nasogastric or gastrostomy tubes may be necessary, with a slow continuous infusion preferred to improve absorption [11]. Patients with SBS and IF usually require long-term PN administered intravenously via a central venous catheter to provide essential macronutrients, micronutrients and fluids, particularly in cases of severe malabsorption or high-output stomas [12,13]. However, long-term intravenous parenteral nutrition is associated with significant complications, including central venous catheter–related bloodstream infections, catheter thrombosis, intestinal failure-associated liver disease (IFALD), metabolic bone disease and metabolic disturbances such as hyperglycemia, electrolyte imbalances, and hypertriglyceridemia [14,15,16]. These complications are not uncommon and contribute substantially to morbidity in this patient population. Catheter-related bloodstream infections have been reported to occur at a mean rate of 0.31 per catheter-year, and parenteral nutrition-associated liver or biliary disorders have been observed in approximately 53% of adult patients after two years of therapy [17,18].

Medical therapies have been used to optimize absorption and reduce symptoms. Antidiarrheal agents (e.g., loperamide, diphenoxylate-atropine) and antisecretory drugs (e.g., proton pump inhibitors) are commonly employed to slow transit and reduce stool output [19]. In recent years, the use of the GLP-2 analog teduglutide (REVESTIVE^®^) has revolutionized the management of SBS by enhancing intestinal adaptation. Teduglutide increases villus height, crypt depth, and mucosal surface area, thereby improving nutrient absorption and reducing dependence on PN. It is administered as a daily subcutaneous injection at a dose of 0.05 mg/kg/day [20].

In randomized placebo-controlled trials, teduglutide has been associated with clinically meaningful reductions in parenteral support requirements, with approximately 63% of treated patients achieving at least a 20% reduction in parenteral support volume at 24 weeks and 68% at 52 weeks, while a subset of patients achieved complete independence from parenteral nutrition [21,22].

Here, we present a non-interventional, multicentre, retrospective cohort study of adult patients with SBS and IF from Greece. This study constitutes a case series, complemented by a narrative review of the current literature. We evaluated the basic clinical and demographic characteristics of these patients, recording and analysing data on the real-world effectiveness of teduglutide, its impact on parenteral nutrition requirements over time and its safety profile, placing our findings in the existing evidence.

## 2. Patients and Methods

### 2.1. Study Design

This was a retrospective, multicenter study involving 5 centers. The study adhered to the Strengthening the Reporting of Observational Studies in Epidemiology (STROBE) guidelines [23], and was approved by the Ethics Committee of University Hospital of Ioannina (Approval No. 25 October 2023/889). Patient anonymity was maintained, and all data were de-identified prior to analysis.

### 2.2. Patients

All eligible patients treated with teduglutide during the study period were included at each participating center. Uniform inclusion and exclusion criteria were applied across centers to ensure consistency in patient selection.

### 2.3. Inclusion Criteria

1. Adult patients (≥18 years) with a diagnosis of SBS who received treatment with teduglutide and had available data before and during therapy were included in the study. Specifically, treatment was considered in adult patients who remained dependent on parenteral nutrition and/or intravenous fluids despite optimized standard medical and nutritional management.
2. A minimum follow-up duration of 52 weeks after initiation of teduglutide therapy.

### 2.4. Exclusion Criteria

Patients who were not receiving PN at the time of teduglutide initiation or those with incomplete or insufficient medical records for outcome evaluation were excluded.

### 2.5. Data Collection

Patients who met the inclusion criteria were identified retrospectively. The following variables were extracted:

1. Demographic and clinical characteristics: age, sex, underlying cause of SBS, duration of SBS prior to initiation of teduglutide, presence or absence of a stoma and stoma type, and body weight and body mass index (BMI) before and after treatment initiation.
2. Laboratory parameters during teduglutide treatment: serum albumin and serum creatinine levels.
3. Parenteral support parameters: total parenteral calorie intake per day and volume of intravenous hydration per week.
4. Teduglutide treatment: administered dose and frequency.
5. Adverse events: type and timing. Adverse events were recorded based on documentation in the medical records and were retrospectively classified according to the Common Terminology Criteria for Adverse Events (CTCAE) v.5.0, based on the available clinical information. According to CTCAE definitions, grade 1 events were considered mild (asymptomatic or mild symptoms not requiring intervention), grade 2 events moderate (symptoms requiring minimal, local, or noninvasive intervention and limiting instrumental activities of daily living), grade 3 severe, grade 4 life-threatening, and grade 5 fatal.

All measurements were recorded at baseline (week 0) and at weeks 4, 12, 26, and 52 of treatment. Data were collected from both electronic and physical medical records and entered into an electronic database created specifically for the purposes of this study. Adjustments in parenteral nutrition and intravenous hydration were made according to routine clinical practice at each center, based on individual patient response and clinical judgment.

### 2.6. Outcomes

The primary outcome of the study was the effectiveness of teduglutide treatment, as measured by changes in parenteral support requirements over time. This was defined as a reduction in total parenteral caloric intake per week and/or a reduction in the volume of intravenous hydration, compared with baseline levels. No predefined percentage reduction threshold was established to define treatment response, given the descriptive and retrospective design of the study. The secondary outcomes included changes in body weight during teduglutide treatment compared with baseline. Changes in laboratory parameters reflecting nutritional and renal status were also assessed, specifically serum albumin and creatinine levels over the course of treatment. The type, timing and management of any adverse events occurring during teduglutide therapy were also recorded. All outcomes were predefined before data extraction and analysis.

### 2.7. Statistical Analysis

All continuous variables were summarised using the mean, standard deviation (SD), median, interquartile range (IQR) and minimum and maximum values. All categorical variables were presented as absolute and relative frequencies. Continuous variables over time are presented as mean values, calculated based on available data at each assessment time point.

## 3. Results

### 3.1. Patients’ Baseline Characteristics

A total of eight adult patients with SBS and IF who received teduglutide treatment were included in the study. All patients received teduglutide at the approved dose of 0.05 mg/kg/day throughout the 52-week follow-up period. Baseline demographic and clinical characteristics are summarized in Table 1. The study population consisted predominantly of female patients (6/8, 75%) and the median age at teduglutide initiation was 53 years. Regarding the anatomical classification of SBS, the majority of patients had type 1 SBS (end-jejunostomy) (n = 6) and two patients had type 2 SBS (jejunocolic anastomosis). Accordingly, the majority of patients had a jejunostomy stoma at baseline. The underlying causes of SBS were heterogeneous. Mesenteric infarction and Crohn’s disease were the most frequent etiologies (2/8 each, 25%), whereas radiation enteritis, neoplasms, abdominal trauma, and intestinal volvulus were each observed in one patient (12.5% each). The median duration of SBS prior to the initiation of teduglutide therapy was 14.5 months. The baseline nutritional status of the cohort was compromised, with a median BMI of 19 kg/m^2^ (IQR 4; range 14.5–20.3), indicating that the majority of patients were underweight or at the lower limit of normal BMI. At baseline, patients required significant parenteral support, with a median weekly intravenous hydration of 5000 mL and a median parenteral nutrition intake of 1500 kcal per day.

### 3.2. Effectiveness

**Figure 1:**
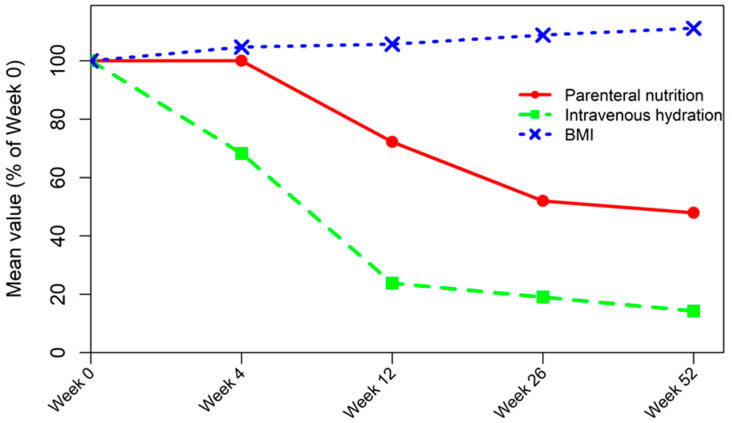
Mean values over time expressed as a percentage of baseline (Week 0). Mean parenteral nutrition, intravenous hydration and BMI values are shown at each follow-up time point, normalized to Week 0.

The primary outcome of the study was to evaluate changes in parenteral support requirements over time during teduglutide treatment, including changes in total parenteral caloric intake and intravenous hydration volume. Teduglutide treatment resulted in a sustained reduction in parenteral nutrition administration. Mean parenteral nutrition calories per day decreased progressively from baseline to Week 52, as detailed in Table 2. During the first 4 weeks of treatment, parenteral nutrition administration remained stable. Mean parenteral nutrition requirements decreased progressively from 1430 kcal/day at baseline to 1430 kcal/day at Week 4, 1125 kcal/day at Week 12, 855 kcal/day at Week 26, and 788 kcal/day at Week 52. Overall, within 52 weeks, there was an approximately 45% reduction in mean parenteral nutrition requirements compared with baseline (Figure 1). Notably, two out of eight patients (25%) achieved complete independence from parenteral nutrition by Week 52; one patient had SBS Type 2 with a jejunocolic anastomosis, while the other had SBS Type 1 with a jejunostomy.

A similar reduction was observed in intravenous hydration administration over the course of treatment. The mean volume of intravenous hydration per week decreased from 5170 mL at baseline to 4000 mL by Week 4, with an even greater reduction by Week 12 (1667 mL/week). This downward progression continued at Weeks 26 (1330 mL/week) and 52 (1000 mL/week), demonstrating a consistent decrease in the administration of intravenous fluids throughout the 52-week study (Table 2). Overall, this corresponded to an approximately 80% reduction in mean intravenous hydration requirements between baseline and Week 52 (Figure 1). Importantly, three out of eight patients (37.5%) had completely discontinued intravenous hydration by Week 52.

BMI increased progressively during teduglutide treatment, as shown in Table 2. Overall, mean BMI increased by approximately 10.6% from baseline to Week 52 (Figure 1). Notably, the most pronounced increase in BMI occurred during the first 4 weeks of treatment, a period during which parenteral nutrition administration remained stable. This early improvement may reflect the rapid intestinotrophic effects of teduglutide, which enhance mucosal growth, increase villus height, and improve absorptive capacity soon after treatment initiation. In addition to improved nutrient absorption, enhanced fluid absorption may have contributed to better hydration status, particularly in patients with chronic subclinical dehydration. Restoration of intravascular and extracellular volume could partly explain the early increase in body weight and BMI. Serum albumin levels showed a modest numerical increase over the course of treatment, which may suggest an improvement in nutritional status. Also, serum creatinine levels showed a gradual decrease, suggesting stable or improved renal function during teduglutide therapy (Table 2).

### 3.3. Safety Profile

Teduglutide treatment was generally well tolerated. Mild adverse events occurred in 3 of 8 patients (37.5%). Two patients reported mild postprandial periumbilical abdominal pain, typically lasting approximately one hour, which resolved spontaneously within 3–4 weeks of treatment initiation. According to CTCAE, abdominal pain is classified as grade 1 when the pain is mild and does not limit activities of daily living. In our patients, the pain was transient, mild in intensity, and did not interfere with daily activities, therefore meeting the criteria for CTCAE grade 1. One patient experienced nausea accompanied by two episodes of vomiting during the first week of therapy; nausea persisted intermittently and was managed with as-needed antiemetic medication. According to CTCAE definitions, vomiting is considered grade 1 when no medical intervention is required. In this case, the vomiting episodes were self-limited and did not require intravenous hydration, enteral or parenteral nutritional support, or hospitalization, and were therefore classified as CTCAE grade 1.

### 3.4. Literature Research

To contextualize the findings of the present case series, a narrative review of the literature on teduglutide in adult patients with SBS and chronic intestinal failure was conducted.

We performed an in-depth review of the literature in PubMed for articles published in English up to December 2025, using the following search string (“short bowel syndrome” OR “intestinal failure”) AND (“teduglutide” OR “GLP-2 analogue”).

Given the narrative design of this review component, a formal systematic review process was not applied. Priority was given to randomized controlled trials, long-term extension studies, meta-analyses, registry-based studies, and large observational cohorts involving adult populations. Pediatric studies were considered selectively when providing important safety data. Case reports and small case series were included when they contributed clinically relevant safety or mechanistic insights.

## 4. Discussion

### 4.1. Mechanisms of Action and Indications of Teduglutide

Teduglutide is a recombinant analogue of human glucagon-like peptide-2 (GLP-2), a hormone that regulates growth, proliferation and maintenance of intestinal epithelial cells. GLP-2 is produced by enteroendocrine L cells located primarily in the distal small intestine and colon. These cells synthesize GLP-2 through tissue-specific post-translational processing of the proglucagon gene, and secretion is stimulated by nutrient intake, especially carbohydrates and fats [24,25]. In SBS, especially following extensive resection of the ileum and/or colon, endogenous GLP-2 secretion is markedly diminished due to loss of L cell mass, resulting in a blunted postprandial GLP-2 response [26,27]. This deficiency limits the adaptive hyperplasia and functional improvement of the remnant intestine, contributing to ongoing dependence on parenteral nutrition and poor enteral autonomy [28].

Teduglutide binds to GLP2 receptors in the intestinal mucosa, stimulating mucosal growth (increasing villus height and crypt depth), enhances intestinal barrier function and slows gastric motility. Together, these effects increase the absorptive capacity of the remaining intestine in patients with SBS and IF, improving absorption of fluids, electrolytes and nutrients, thereby reducing the need for parenteral support (parenteral nutrition and/or intravenous fluids) in patients with SBS [20,29,30]. Owing to teduglutide’s resistance to in vivo degradation by dipeptidyl peptidase IV, it has a longer half-life than native GLP-2 (1.3 h vs. 7 min) [31]. Teduglutide is indicated for the treatment of SBS in patients with chronic intestinal failure who are dependent on parenteral support (parenteral nutrition and/or intravenous fluids), and who are unable to maintain metabolic homeostasis through oral or enteral intake alone. This includes both adults and children aged ≥1 year, as approved in the United States, Europe and Japan. Teduglutide is not indicated for SBS patients who are not dependent on parenteral support [32,33].

### 4.2. Clinical Trial and Real-World Evidence for Teduglutide in Short Bowel Syndrome

Randomized clinical trials and real-world cohorts have demonstrated the effectiveness of teduglutide in patients with SBS. In a randomized, placebo-controlled clinical trial of 24 weeks, teduglutide significantly reduced parenteral support requirements compared with placebo. A ≥20% reduction in parenteral support volume was achieved by 63% of teduglutide-treated patients versus 30% in the placebo group (*p* = 0.002). At week 24, mean weekly parenteral support volume decreased by 4.4 ± 3.8 L with teduglutide compared with 2.3 ± 2.7 L with placebo (*p* < 0.001). Additionally, a reduction of at least one infusion day per week occurred more frequently in the teduglutide group (54% vs. 23%; *p* = 0.005) [22]. Similarly, in a subsequent randomized clinical trial with a double-blind extension, teduglutide demonstrated sustained effectiveness in reducing parenteral nutrition requirements over 52 weeks. Progressive reductions in parenteral nutrition volume were observed with both tested doses. By week 52, a clinically meaningful ≥20% reduction in weekly parenteral nutrition volume was achieved by 68% of patients receiving 0.05 mg/kg/day and 52% of those receiving 0.10 mg/kg/day. A reduction of at least one parenteral nutrition infusion day per week occurred in 68% and 37% of patients, respectively, supporting the long-term effectiveness of teduglutide [21]. A meta-analysis of ten studies was conducted to evaluate the effectiveness of teduglutide in adults with short bowel syndrome who are dependent on parenteral support. The pooled response rate (defined as a ≥20% reduction in parenteral support) increased over time, reaching 64% after 6 months, 77% after 1 year, and 82% after ≥2 years. Parenteral support independence was achieved by 11%, 17% and 21% of patients at these time points, respectively. Having a continuous colon was associated with a lower response rate but a higher likelihood of weaning from parenteral support. SBS aetiology did not significantly influence outcomes. These findings provide strong evidence supporting the long-term effectiveness of teduglutide in adults with SBS [20]. In Japanese adults with SBS and intestinal failure, teduglutide treatment in 18 patients was associated with clinically meaningful and sustained reductions in parenteral support requirements, with ≥20% volume reductions observed at 24 weeks and in all 11 patients with long-term follow-up up to 4.5 years. Mean parenteral support volume decreased progressively over time, reaching an approximate 57% reduction at the interim data cut-off [34]. In a post hoc analysis of a phase III trial including 85 patients with SBS and intestinal failure, teduglutide was associated with significant reductions in parenteral support volume, strongly correlated with baseline parenteral support requirements. The largest reductions were observed in patients with jejunostomy or ileostomy, who experienced significantly greater volume decreases than both placebo-treated patients and teduglutide-treated patients with colon in continuity, while intermediate effects were seen in other bowel anatomies [35]. Evidence also suggests that teduglutide may be an effective therapeutic option for patients with Crohn’s disease and SBS-associated intestinal failure, reducing parenteral support requirements without adversely affecting the course of the underlying disease. In a retrospective cohort of 32 adults with Crohn’s disease-associated chronic intestinal failure and SBS, teduglutide was associated with significant clinical improvement. A ≥20% reduction in parenteral support was achieved by 26 of 32 patients, accompanied by marked reductions in parenteral nutrition use, weekly volume, and infusion frequency. Improvements were also observed in stool output, diarrhea control, and patient-reported symptoms, with reduced reliance on antidiarrheal medications, while immunosuppressive therapy remained unchanged [36].

Another retrospective study involved 18 patients with Crohn’s disease-associated short bowel syndrome and intestinal failure who were treated with teduglutide. Two patients (11%) discontinued treatment due to intolerance, while 16 continued. Teduglutide significantly reduced the volume of parenteral support from 15,825 to 10,700 mL per week by week 8 (*p* = 0.0038), with 43.8% of patients achieving a reduction of at least 20%. Early reduction in parenteral support occurred only in patients without colon continuity. No serious adverse events occurred [37]. An analysis of a single-center cohort evaluated the real-world eligibility of adults with SBS-associated intestinal failure for treatment with the GLP-2 analogue teduglutide. Seventy-nine patients were categorized as non-candidates, potential candidates, or straight candidates according to approved indications, contraindications, warnings, and phase III trial criteria. Overall, 34.2% were non-candidates due to malignancy risk, recent cancer, or listing for intestinal transplantation, while 30.4% were considered potential candidates because of premalignant conditions, obstruction risk, fistulas, or severe comorbidities. The remaining 35.4% were straight candidates and demonstrated the lowest intravenous supplementation requirements, including fewer infusion days per week and reduced energy and volume needs. This structured approach supports standardized patient selection and facilitates comparison of clinical outcomes [38].

### 4.3. Safety Profile and Long-Term Risks of Teduglutide

The safety profile of teduglutide has been evaluated in both clinical trials and real-world cohorts. Overall, teduglutide is considered well tolerated, with most adverse events being mild to moderate in severity and manageable. The most frequently reported adverse events include gastrointestinal symptoms such as abdominal pain, nausea, vomiting, abdominal distension, and flatulence, as well as upper respiratory tract infections and injection site reactions. The majority of events are consistent with the pharmacodynamic effects of GLP-2 on intestinal motility, secretion, and mucosal growth [39,40]. In a randomized placebo-controlled trial in adult patients with SBS receiving the dose of 0.05 or 0.1 mg/kg once daily, the most common adverse events included headache (35%), nausea (31%), and abdominal pain (25%) [21,41]. In pediatric populations, pooled safety data from four clinical studies demonstrated that vomiting (51.7%), pyrexia (43.8%), upper respiratory tract infection (41.6%) and cough (33.7%) were the most common. 39.3% of the adverse events were considered to be related to teduglutide treatment, with abdominal pain and vomiting being the most frequent (5.6% each) [39]. Real-world data are consistent with these findings; a large U.S. database study including 170 patients reported abdominal pain in 41.2% and nausea in 23.5% of patients treated with teduglutide [42]. Although uncommon, cardiac adverse events have been observed during treatment with teduglutide. The incidence of congestive heart failure in adult clinical trials of teduglutide was 3% in teduglutide-treated patients, with two cases reported [41,43]. In pediatric cohorts, heart failure due to left ventricular hypertrophy was reported as a serious adverse event in 1 out of 104 children treated with teduglutide, and was considered possibly related to the drug [44].

Teduglutide has been associated with an increased risk of developing gastrointestinal polyps, including adenomas with low-grade dysplasia, in patients with SBS. Clinical studies and case reports have described the development of de novo polypoid lesions during teduglutide therapy, most commonly hyperplastic polyps and adenomas, with low-grade dysplasia occasionally observed, but no cases of high-grade dysplasia or malignancy reported in surveillance cohorts [45,46]. GLP-2 analogues, such as teduglutide, have intestinotrophic and anti-apoptotic effects, which raise theoretical concerns regarding neoplastic progression, particularly with long-term exposure [47]. The STEPS-2 trial, a two-year open-label extension, evaluated the long-term safety and efficacy of teduglutide in adults with SBS. Among 50 patients who underwent 51 colonoscopies, gastrointestinal polyps were identified in nine cases (five adenomas, one hyperplastic, one inflammatory, and two unclassified), with no evidence of dysplasia or malignancy [48]. A pooled analysis of safety data from four prospective clinical trials of teduglutide (173 patients; 222 person-years; up to 2.5 years of exposure) identified colonic, rectal, and small intestinal polyps in 3 (1.7%), 2 (1.2%), and 1 (0.6%) patients, respectively [40]. In addition, using interim data from a multinational SBS-IF registry (up to 4 years of follow-up), the long-term safety of teduglutide in adult patients with SBS and intestinal failure was evaluated. Long-term safety was compared between patients ever treated with teduglutide and those never exposed to the drug and managed with standard of care. Among 1296 enrolled patients (540 ever-treated; 756 never-treated), no cases of colorectal cancer were observed during follow-up. More colonoscopies were performed in the ever-treated group. New or worsening colorectal polyps occurred more frequently in ever-treated than never-treated patients (13.4 vs. 2.7 per 1000 patient-years), while incidence rates of any malignancy were similar between groups [49]. In this context, colonoscopy, gastroscopy, and abdominal ultrasound should be performed before treatment initiation, after one year, and every 3–5 years thereafter. Pre-existing non-malignant gastrointestinal polyps are not an absolute contraindication but require enhanced monitoring [45,50]. Teduglutide is contraindicated in patients with active gastrointestinal malignancy, and caution is warranted in those with a history of gastrointestinal neoplasia [51].

### 4.4. Limitations

This study has several limitations that should be acknowledged. First, the retrospective design may introduce selection and information bias, and minor adverse events may have been underreported due to the retrospective nature of data collection. Second, no uniform, protocolized parenteral support weaning algorithm was applied across participating centers, and concomitant co-interventions (including dietary modifications, antidiarrheal therapy, or fluid management strategies) were not systematically recorded, which may limit the interpretability of treatment effects. Third, given the small sample size and limited statistical power, formal hypothesis testing and adjusted longitudinal modeling were not performed. Therefore, the findings should be interpreted as descriptive and hypothesis-generating. Moreover, due to the limited sample size, further analyses exploring potential predictors of treatment response (such as disease duration or anatomical subtype) were not feasible. However, SBS-associated IF is a rare condition, and the present study reflects real-world experience across five centers in Greece. Published real-world data on teduglutide use in Greece remain scarce. Although the results cannot be generalized to all SBS populations, the observed trends are consistent with findings from randomized clinical trials and large observational cohorts, supporting the external plausibility of our observations.

## 5. Conclusions

Interpreting the findings of the present case series in conjunction with the available literature further supports the use of teduglutide as an effective and well-tolerated treatment for adult patients with SBS and IF. In our cohort, teduglutide treatment was associated with a progressive and sustained reduction in parenteral nutrition and intravenous fluid requirements over 52 weeks, alongside an improvement in nutritional status as reflected by an increase in body mass index. Importantly, complete independence from parenteral support was achieved in a subset of patients, highlighting the potential of teduglutide, through its intestinotrophic effects, to enhance intestinal adaptation.

These real-world observations are consistent with evidence from randomized controlled trials, extension studies, meta-analyses, and large observational cohorts, which demonstrate durable reductions in parenteral support and, in a subset of patients, complete independence from parenteral nutrition. However, given the absence of a comparator group, the observed improvements should be interpreted as a temporal association rather than definitive evidence of causality. Furthermore, both the literature and our experience confirm a favorable safety profile, with adverse events being predominantly mild, gastrointestinal in nature, and manageable with appropriate monitoring (Box 1). Future prospective, larger-scale studies are warranted to better define predictors of response and to optimize the long-term management of SBS patients treated with teduglutide.

Box 1 **Clinical Considerations for Teduglutide Therapy in Short Bowel Syndrome.**

➢ Teduglutide is an effective therapeutic option for adult patients with short bowel syndrome and chronic intestinal failure, leading to **meaningful and sustained reductions in parenteral nutrition and intravenous fluid requirements**.

 

➢ Long-term treatment with teduglutide may result in **complete independence from parenteral nutrition in a subset of patients**, reflecting enhanced intestinal adaptation mediated by its **intestinotrophic effects**.

 

➢ The safety profile of teduglutide is generally favorable. The **most common adverse events are gastrointestinal**, including abdominal pain, nausea, vomiting, abdominal distension, diarrhea, and flatulence; injection-site reactions and upper respiratory tract also been reported. Most adverse events are **mild to moderate in severity** and manageable.

 

➢ Due to its intestinotrophic and anti-apoptotic properties, teduglutide has been associated with the development of **gastrointestinal polyps**, necessitating structured endoscopic surveillance.

 

➢ **Endoscopic monitoring is recommended**, including baseline colonoscopy and upper gastrointestinal endoscopy prior to treatment initiation, repeat evaluation after one year, and subsequent surveillance at regular intervals.

 

➢ Teduglutide therapy requires **careful patient selection and long-term multidisciplinary follow-up**, integrating clinical, nutritional, laboratory, and endoscopic assessment to optimize efficacy and ensure safety.

## Figures and Tables

**Table 1:** Basic characteristics of the patients.

**Gender**	
**Male, n (%)**	**2 (25%)**
**Female, n (%)**	**6 (75%)**
**Age of patient at teduglutide initiation**	
Median (IQR)	53 (17)
Range	19–71
**Type of SBS**	
Type 1 (SBS-J), n (%)	6 (75%)
Type 2 (SBS-JC), n (%)	2 (25%)
**Cause of SBS, n (%)**	
Mesenteric infarction	2 (25%)
Crohn disease	2 (25%)
Radiation enteritis	1 (12.5%)
Neoplasms	1 (12.5%)
Abdominal trauma	1 (12.5%)
Intestinal volvulus	1 (12.5%)
**Duration of SBS before initiation of teduglutide** (months)	
Median (IQR)	14.5 (45)
Range	2–84
**BMI patient before teduglutide (kg/m^2^)**	
Median (IQR)	19 (4)
Range	14.5–20.3
**Creatinine before teduglutide(mg/dL)**	
Median (IQR)	1.19 (1.2)
Range	0.5–2.2
**Albumin before teduglutide (g/dL)**	
Median (IQR)	3.1 (0.9)
Range	2.3–4.0
**Intravenous hydration per week (mL)**	
Median (IQR)	5000 (4000)
Range	2000–7000
**Parenteral nutrition calories per day**	
Median (IQR)	1500 (750)
Range	800–2000

**Table 2:** Changes in parenteral nutrition requirements, intravenous hydration volume, BMI, and laboratory parameters during 52 weeks of teduglutide treatment (mean ± SD).

	Week 0	Week 4	Week 12	Week 26	Week 52
**Mean PN calories/day (kcal)**	1430	1430	1125	855	788
(SD)	(459)	(459)	(702)	(705)	(685)
**Mean IV hydration (mL/week)**	5170	4000	1667	1330	1000
(SD)	(2228)	(2828)	(2065)	(1632)	(1095)
**Mean BMI (kg/m^2^)**	18.9	19.7	19.9	20.5	20.9
(SD)	(1.7)	(1.3)	(1.4)	(1.9)	(1.6)
**Creatinine (mg/dL)**	1.45	1.40	1.32	1.36	1.28
(SD)	(0.61)	(0.52)	(0.45)	(0.40)	(0.39)
**Albumin (g/dL)**	3.1	3.24	3.4	3.54	3.48
(SD)	(0.61)	(0.48)	(0.37)	(0.55)	(0.35)

## Data Availability

All data generated or analysed during this study are included in this published article.
